# Applying lessons of COVID-19 and other emerging infectious diseases to future outbreaks

**DOI:** 10.1128/mbio.01109-24

**Published:** 2024-05-23

**Authors:** Evan M. Bloch, David J. Sullivan, Arturo Casadevall, Shmuel Shoham, Aaron A. R. Tobian, Kelly Gebo

**Affiliations:** 1 Department of Pathology, Johns Hopkins Hospital, Baltimore, Maryland, USA; 2 Department of Molecular Microbiology and Immunology, Johns Hopkins Bloomberg School of Public Health, Baltimore, Maryland, USA; 3 Department of Medicine, Division of Infectious Diseases, Johns Hopkins University School of Medicine, Baltimore, Maryland, USA; Albert Einstein College of Medicine, Bronx, New York, USA

**Keywords:** communicable diseases, emerging, COVID-19, Mpox (monkeypox), public health, policy

## Abstract

Infectious diseases are emerging and re-emerging far more frequently than many appreciate. In the past two decades alone, there have been numerous outbreaks (e.g., Ebola, chikungunya, Zika, and Mpox) and pandemics (i.e., swine flu and coronavirus disease 2019) with profound effects to public health, the economy, and society at large. Rather than view these in isolation, there are important lessons pertaining to how best to contend with future outbreaks of emerging infectious diseases. Those lessons span definition (i.e., what constitutes a pandemic), through deficiencies in surveillance, data collection and reporting, the execution of research in a rapidly changing environment, the nuances of study design and hierarchy of clinical evidence, triage according to clinical need as supply chains become overwhelmed, and the challenges surrounding forecasting of outbreaks. Understanding those lessons and drawing on both the successes and failures of the past are imperative if we are to overcome the challenges of outbreak/pandemic responsiveness.

## OPINION/HYPOTHESIS

Outbreaks of infectious diseases are occurring more frequently than many appreciate. In the past two decades alone, there have been numerous viral outbreaks (e.g., Middle East respiratory syndrome, Ebola, dengue, chikungunya, Zika, Mpox [formerly monkeypox]) and two pandemics (i.e., swine flu and coronavirus disease 2019 [COVID-19]), with profound effects on health, the economy, and society. While it is easy to focus on the missteps of each of these public health crises, they have yielded many positive developments. The proliferation of molecular testing will prove invaluable to new epidemics, with improved diagnostics, real time surveillance, and linkage to care. Likewise, there is improved communication from public health agencies, engaging a wider array of stakeholders. During the COVID-19 pandemic, federal agencies, academic institutions, and industries collaborated closely, expediting approval of technologies, vaccines, and therapies. The unprecedented speed of development and distribution of novel, highly effective vaccines and treatments ultimately proved to be central to the control of the pandemic. Numerous clinical trials were executed successfully under enormously challenging conditions. Preprint expedited the dissemination of information, avoiding the prior languid timelines to formal publication and clinical adoption. There have also been behavioral changes, given greater education regarding infectious diseases and the available preventive measures, such as hygiene, masking, and social distancing. For the next public health crisis, deliberate efforts to avoid the mistakes of the past are critical.

While emerging infectious diseases (EIDs) share similarities, modes of transmission, lethality, and populations at risk vary. Severe acute respiratory syndrome coronavirus 2 (SARS-CoV-2) (COVID-19) and H1N1 influenza virus (swine flu) are respiratory pathogens accounting for their scale and rapidity of spread. By contrast, Ebola and Mpox virus are spread primarily through direct contact, each posing formidable—albeit different—challenges: Ebola for its high infectivity, requiring barrier methods, often in austere environments (e.g., in Central and West Africa) and Mpox for its association with men who have sex with men (MSM). Emergence in any minority group poses risk of stigmatization, which interferes with public health efforts (e.g., contact tracing and treatment). This requires concerted effort to minimize stigmatization while still addressing the escalation in incidence, through targeted provision of resources and education to affected groups (1).

Surveillance is lacking, particularly in locations where EIDs are most likely to originate. Once outbreaks occur, forecasting their trajectory is enormously complicated. With COVID-19, there was near-constant change from the rampant spread of the early pandemic to intermittent outbreaks coinciding with the emergence of variants and subvariants. In parallel, there were continually shifting policies amid the advent of new diagnostic, preventive, and therapeutic interventions.

Definitions remain vague regarding what constitutes a public health emergency or pandemic. Designation as a pandemic would motivate for allocation of resources, yet a premature declaration has broad ramifications for daily life. Conversely, a moderated classification may slow the response when intervention is needed most. The response to EIDs in recent years suggests that designation of outbreaks is not uniform and that political and social factors are impacting the associated public health response. Per current definition, “pandemic refers to an epidemic that has spread over several countries or continents, usually affecting a large number of people” (2). There were 28,600 cases and 11,325 deaths of Ebola in 10 countries in the 2014–2016 outbreak (3). The World Health Organization declared a “public health emergency of international concern (PHEIC) (4), which is designated only for events with a risk of potential international spread or that require a coordinated international response” (3); the Ebola outbreak was never escalated to pandemic. Mpox, another PHEIC, was never designated as a sexually transmitted infection despite sexual contact being the major mode of transmission in its largest outbreak to date (5, 6). If these definitions were applied stringently, the scale of some of the EIDs of the last decade, notably Zika, Ebola, and Mpox, might have been lessened through more timely mobilization of resources. In short, concerns over how policies are perceived may be at odds with good science, thus frustrating public health efforts.

Testing is critical in outbreak responsiveness. A proactive response relies on the development of tools (i.e., production of assays) against a threat that has yet to be realized. This raises the question as to how one incentivizes investment for diagnostics and therapeutics for a market that is yet to materialize. There are pathogens (e.g., Oropouche and Mayaro) that are well positioned for epidemic spread, yet these lack high-throughput assays for surveillance or diagnosis (7).

Harmonization of effort was central to many of the missteps in COVID-19. Numerous studies, spanning multiple funding agencies, replicated each other in design, target population, and—in many cases—their results. Initial COVID-19 therapeutic research was focused, disproportionately, on hospitalized patients with severe disease (8–10). From 1 January 2020 to 26 October 2022, a total of 39 inpatient clinical trials and 70 matched cohort studies were conducted to evaluate the efficacy of COVID-19 convalescent plasma (CCP) (11). CCP was shown repeatedly to be ineffective in unselected hospitalized patients with moderate to severe COVID-19. By contrast, few studies of CCP were conducted in early disease (e.g., an outpatient setting), in which it was shown to be beneficial, preventing hospitalization (12).

The research apparatus is not designed for the acuity of need encountered in outbreak situations. For much of the COVID-19 pandemic, efforts were haphazard, stalled by *ad hoc* funding streams and administrative barriers, leaving a host of foundational questions unanswered. Even under the best of circumstances, the research cycle is measured in months to years. Research that is initiated in times of outbreak is challenging, amid parallel scaling of interventions, changes in policy, and standards of care, which collectively impact subjects’ eligibility for enrollment into planned or active studies, thus risking shortfall in recruitment, leaving studies underpowered to demonstrate their outcomes (13, 14).

The role of randomized control trials (RCT) in the response to EIDs needs to be carefully considered. RCTs are regarded as the gold standard of evidence. However, without a thorough understanding of the condition being studied and how it impacts important subpopulations, RCTs can lead to erroneous conclusions (15, 16). Moreover, the cost and complexity of executing RCTs are high, frequently requiring sponsorship from for-profit pharmaceutical companies. Although well positioned to bring new drugs to market, these companies put stringent limits on the types of studies that they are willing to support. For example, immunocompromised patients were disproportionately impacted by the COVID-19 pandemic yet comprised a very small percentage of participants in RCTs. At the other extreme, immunocompetent patients with broad immunity against SARS-CoV-2 (e.g., through natural infection and/or vaccination) are also understudied.

Observational studies are often devalued despite their seminal contributions to the field of infectious diseases. For example, a case series of individuals with *Pneumocystis jiroveci* pneumonia and Kaposi’s sarcoma in MSM triggered investigation and recognition of the HIV/AIDS crisis; three patients with hemophilia highlighted the hazards of transfusion-transmitted HIV (17, 18). Early administration of combination antiretroviral therapy for HIV was informed by observational data. Supporting trial evidence for this practice would arrive over three decades following the start of the HIV/AIDS pandemic (19). Of note, most therapies and medical practices have never been subjected to clinical trial evaluation.

Clinical trials are not without their caveats (20). Rigid adherence to study design in clinical trials has found its way into lore, with pre-specified primary outcome measures assuming absolute importance. Emphasis on faithfulness to a primary outcome is to dissuade investigators from trawling blindly for random findings. While the intent of that position is sound, it comes at the peril of disregarded or diminished secondary findings (e.g., return to work, resolution of symptoms, and/or viral load reduction) that could otherwise prove to be significant. Separately, clinical trial findings are often extrapolated, well beyond the bounds of what the studies were intended to accomplish and generalized to populations that were not studied. This becomes problematic as a well-executed clinical trial of a given intervention—albeit in the wrong study population—risks a negative finding and blanket designation of futility (e.g., efficacy of influenza convalescent plasma [CP] and monoclonal antibodies for COVID-19 in hospitalized patients) (21).

During outbreaks, demand often outstrips supply. In the example of the 2022 Mpox outbreak, oral antiviral agents and Mpox vaccines were available at the start of the outbreak (unlike in the case of COVID-19). Nonetheless, the supply was rapidly overwhelmed by the unprecedented demand. The science and strategy of vaccination were stalled by the physical challenges of packaging vials, sourcing the necessary materials to do so, or having adequate staffing for administration. There were insufficient vaccine doses to meet the needs of the populations at risk, culminating in public health emergencies in selected cities such as San Francisco and New York. The situation forced a salvage approach to increase the coverage with the available supply. For example, there was a tiered allocation of vaccines to those at highest risk and/or opting for vaccination through a single—rather than the preferred double dose—regimen and dosing of the vaccine through intradermal methods to increase the number of vaccines that could be given per dose (22). In parallel, there were efforts to improve the supply chain. The vaccine bottleneck was reminiscent of the initial phase of the COVID-19 vaccination program, barely 2 years previously.

Biological plausibility may be ignored amidst the panic of the pandemic response: an aggressive response still needs to uphold sound scientific principle. Guided by minimal data, some therapies such as hydroxychloroquine, an antimalarial, and ivermectin, an anthelminthic, were re-purposed and distributed en masse during the COVID-19 pandemic, ignoring biological plausibility and serving as a distraction. Instead, initial attempts at treatment must focus on those therapies that are most likely to be safe and effective.

Conditions also change during epidemics, requiring continual re-evaluation of plausibility. For example, nearly the entire U.S. population now possesses high-level immunity against SARS-CoV-2. Nonetheless, drugs such as nirmatrelvir/ritonavir, which gained regulatory approval based on data gleaned from largely non-SARS-CoV-2 immune individuals, continue to be marketed for patients who may no longer derive benefit. Additionally, one can no longer assume that scientific recommendations will be accepted, which—in part—may be ascribed to erosion of trust in the medical community. Unfortunately, ease of communication (e.g., social media) has allowed misinformation to flourish. Similarly, politicization of science, including among members of the medical community, is formidable.

The recurrent déjà vu pertaining to EIDs demands proactive solutions. Foremost is improved surveillance with expansion of networks that target human (e.g., blood donors) and animal populations, proximal to areas where outbreaks are most likely to occur (i.e., strengthening capacity in low- and middle-income countries). Innovative, low-cost strategies have long been in effect for arboviruses (e.g., seasonal testing of dedicated flocks of “sentinel” chickens for West Nile virus in the United States), enabling early detection of outbreaks (23). There are also underutilized technologies (e.g., metagenomics, multi-agent serological profiling) that could enhance the ability to detect EIDs in a timely manner. Reporting to public health agencies is critical to surveillance and requires improved communication between data systems, ideally with automated capture of notifiable information.

Second, executive function needs to bypass normal procedure when the circumstances demand a rapid and deliberate response. There are models that matched the pace of intervention to the scale of threat successfully. Operation Warp Speed, a U.S. government-led initiative, promoted public–private partnership, thus enabling stakeholders to evade many of the administrative hurdles that typically slow research and development (i.e., in this case focused on diagnostics, vaccines, and therapies for COVID-19). Funds were committed prior to knowledge of efficacy of the supported initiatives. This highlighted the benefits of public–private partnership, across government, academic institutions, and industry.

The third solution pertains to research: areas for improvement span the entire continuum from funding to study execution, interpretation, and reporting of results. In times of outbreak, research consortia need to be activated early, with better assignment of roles to avoid duplication of efforts and to ensure that the breadth of questions is addressed. Funding agencies encourage investigators to submit proposals for supplementary funding requests; this serves to streamline support for ancillary studies that are needed early in outbreaks. Rather than a passive process that depends on investigator initiatives, funding agencies could direct investigators to pursue relevant projects. There are numerous examples of large, international networks (e.g., HIV Prevention Trials Network) with robust infrastructure for clinical and laboratory-based studies that could be leveraged in times of need. Pre-existing protocols and materials from active or prior studies could be re-purposed rapidly and efficiently, such as the adaptation of dengue protocols to study emerging arboviruses.

Clinical trials are logistically complex during outbreaks and pandemics. As such, RCTs require careful consideration of the target population. Registries could provide critical early information for the design of RCTs (24). While tempting to focus on those who are most severely affected (e.g., advanced COVID-19), those same patients are often less likely to benefit from a given therapy, thus masking effect. Instead, those with early disease may benefit more from a given intervention. Study design must ensure diverse enrollment and representation as there may be differential effects of a given intervention and different reasons for participation or non-participation. The near constant change of research of EIDs requires adaptability to ensure that investigators can pivot, thus salvaging studies and optimizing their gains.

Fourth, policy should consider the individual pathogens. Vaccine programs offer a good illustration of this principle. Decentralization of vaccination programs enables local groups to vaccinate those at highest risk: Mpox, respiratory syncytial virus, and measles are three pathogens where this would be appropriate (i.e., for MSM, those of advanced age, and the unvaccinated, respectively). By contrast, a centralized model allocates vaccine quotas proportionately, independent of risk. This is appropriate when confronted by a pathogen that impacts the majority of a given population. When resources such as vaccines are in short supply, one needs to consider which model is most appropriate. Early in the COVID-19 pandemic, micromanagement of vaccination based on stipulated high-risk groups was counterproductive. Many were left waiting to be vaccinated, while some who were eligible remained unwilling to accept vaccination. In the case of Mpox, vaccine manufacture could have been decentralized earlier, with a view to increase the supply. Vaccination also highlights the ethical concerns surrounding the prioritization of patient populations when there is insufficient supply to meet demand. In the United States, there was phased distribution of COVID-19 vaccines favoring those of advanced age and/or with co-morbid risk factors for severe disease. Vaccination for Mpox prioritized MSM and laboratory staff. Clinical healthcare workers were not included, at least not initially (this policy was later amended largely due to advocacy by the Centers for Disease Control and Prevention).

Healthcare providers should be prioritized, given their voluntary exposure to risk, in much the same way that emergency responders and firefighters are instructed—explicitly—not to assume risk without appropriate protection.

In conclusion, the myriad of EIDs over the past two decades, culminating in the historic COVID-19 pandemic, has illuminated the scale of threat to public health. These perennial health emergencies are enormously instructive as to how best to respond. It is imperative to heed the lessons of yesterday if to improve upon tomorrow.
